# Sterile necrotizing and non-necrotizing granulomas in a heart transplant patient with history of PTLD: A unique finding

**DOI:** 10.1016/j.ijscr.2019.05.054

**Published:** 2019-06-04

**Authors:** Nicholas Piening, Saxena Saurabh, Armando Salim Munoz Abraham, Hector Osei, Colleen Fitzpatrick, Jose Greenspon

**Affiliations:** a Department of Pediatric Surgery, Cardinal Glennon Children’s Medical Center, United States; b Saint Louis University School of Medicine, United States

**Keywords:** Case report, Pediatric surgery, General surgery, Transplant surgery, Posttransplant lymphoproliferative disease

## Abstract

**Introduction:**

Posttransplant lymphoproliferative disease (PTLD) is a known complication in patients with solid organ transplant. It can present as localized or disseminated tumor. The cornerstone of management consists of reduced immunosuppression (RI). In select cases, localized disease can potentially be curative with surgical excision.

**Presentation of case:**

Here we present a case of a 19-year-old female with orthotopic heart transplant with two episodes of recurrent PTLD. After the second episode she was found to have asymptomatic splenic lesions which were refractory to RI and chemotherapy. She subsequently underwent splenectomy that showed sterile necrotizing and non-necrotizing granulomas with no evidence of PTLD.

**Discussion:**

Based on our literature search this is the first ever reported case of sterile granulomas in a patient with recurrent PTLD which could potentially be diagnosed with minimally invasive biopsy rather than diagnostic splenectomy.

**Conclusion:**

This report is an attempt to create awareness regarding potential for presence of sterile granulomas in patients with recurrent PTLD and discuss the use of percutaneous biopsy before splenectomy.

## Highlights

• This is the first ever reported case of sterile granulomas in a patient with PTLD.
• PTLD is a disease that could potentially be diagnosed with minimally invasive biopsy rather than diagnostic splenectomy.
• This report is to create awareness regarding potential presence of sterile granulomas and discuss use of biopsy before splenectomy.

## 1 Introduction

Granulomas are focal collection of inflammatory cells that are usually the result of chronic inflammation. Granulomatous inflammation is a common pathologic finding that can be caused by a variety of conditions including infection, autoimmune, allergic, drug or neoplasm. The majority of granulomas can be explained by infectious etiology, however, it has been reported in the literature that between 25–39% are sterile and the etiology remains unknown despite clinical, serological and microbiological evaluation [1]. It is hypothesized that in cases of sterile granulomas, microorganisms have been killed and/or removed by inflammatory processes [2]. Given this hypothesis, it would be very unexpected and unusual to discover sterile granulomas in an immunocompromised patient such as patients that receive organ transplants and are on immunosuppressive medications.

While the majority of necrotizing granulomas are infectious in etiology, about 25% of these findings remain unexplained [3]. However, these cases are all documented in non-transplant patients with robust immune systems capable of inflammatory processes to kill and/or remove microorganisms [2]. In review of the current literature, there have been no cases of granulomatous inflammation without organisms in patients with post-transplant lymphoproliferative disorder (PTLD). Therefore, the presence of sterile granulomas in our patient with history of recurrent PTLD is a rare finding. The overall prevalence of Epstein-Barr virus (EBV)-positive PTLD following solid organ transplant is variable, ranging from 1% to 20%. These variabilities depend on the type of solid organ transplanted, pretransplant EBV serostatus and the age of the recipient [[4], [5], [6]].

There are a handful of Epstein-Barr Virus (EBV) negative PTLD documented in the literature [7,8]. A wide spectrum of morphology can be observed in EBV-negative PTLD lesions ranging from nonspecific reactive hyperplasia to large-cell lymphoma. Of the 11 documented cases by Leblond, three contained polymorphic morphology and the remaining eight consisted of monomorphic morphology. However all of these contained centroblasts and/or immunoblasts with plasmacytic differentiation meeting the criteria for Diffuse Large B-Cell Lymphoma (D-LBCL) [9]. In a case series done by a single center, three cases of EBV-negative PTLD was found and morphology showed the following: monomorphic lymphocytes with plasma cells indicating mucosa-associated lymphoid tissue (MALT) lymphoma, D-LBCL and atypical Burkitt lymphoma [8]. Additionally, a systematic review performed looking for EBV-negative PTLD cases found 17 patients and 26 specimens that qualified for the study. Of these 26 specimens the following pathologies were found: infectious mono-like PTLD, polymorphic PTLD, monomorphic-(large noncleaved (centroblastic)-like) PTLD, monomorphic-(small noncleaved-like) PTLD, monomorphic- (Immunoblastic B cell-like) PTLD, medullary plasmacytosis PTLD, small B-cell like neoplasm and medullary plasmacytosis large granular lymphocyte disorder [7].

Upon review of the current literature, granulomatous inflammation without organism s in patients with PTLD is not common. There are currently no documented cases of disease negative PTLD with morphology consistent with multifocal granulomas. We report the case of a 19-year-old female that underwent heart transplant complicated with development of recurrent PTLD with the finding of three sterile granulomas. Our work has been reported in line with the SCARE criteria [10].

## 2 Presentation of case

Our patient is a 19-year-old female with a complex past medical history significant for muscular dystrophy with restrictive cardiomyopathy requiring cardiac transplant in 2010. Her post transplant course was complicated by recurrent episodes of PTLD.

The first incidence of PTLD was found five years after the transplant when she presented with chest pain and shortness of breath. Computed tomography (CT) showed bilateral apical blebs for which she underwent open left thoracotomy with resection of blebs and pleurodesis. Pathologic evaluation showed polymorphic PTLD that was strongly EBV positive. She was treated successfully with chemotherapy and steroids as her follow-up CT showed resolution of lesions with EBV negative polymerase chain reaction (PCR).

A recurrence of PTLD was discovered one year after the first PTLD diagnosis. During that admission the patient presented with fever, chills, nausea, vomiting and hypotension. Renal ultrasound was ordered due to concern for pyelonephritis which showed multiple hypoechoic lesions in spleen concerning for recurrent PTLD although EBV PCR was lower than previous instance of PTLD.

CT with intravenous contrast of the neck, chest, abdomen and pelvis showed multiple subpleural cysts, ill-defined hypoattenuating lesions in the spleen, enlarged spleen, moderate volume ascites in the pelvis and enlarged left inguinal lymph node. These findings were not present on previous CT scan discussed above.

Given these new findings and the history of heart transplant and immunosuppression, surgery was consulted for biopsy and gastrostomy tube placement due to poor oral intake. She underwent left inguinal lymph node biopsy and laparoscopic gastrostomy tube placement for nutrition.

Pathology showed recurrent monomorphic PTLD with EBV positive for which she underwent three cycles of chemotherapy. Flow cytometry did not show any evidence of non-Hodgkin lymphoma.

Four weeks after completing chemotherapy, follow-up positron emission tomography (PET)/CT scan showed numerous fluorodeoxyglucose avid lesions in the spleen and right inguinal lymph nodes suspicious for malignancy. She was then referred to pediatric surgery for evaluation for lymph node biopsy and possible splenectomy.

On initial examination the abdomen was soft, non-tender, and the spleen was not palpable. The gastric tube was in place and functional. Blood work-up showed hemoglobin of 11.4 g/dL (12.1–15.1), decreased lymphocytes and increased monocyte and eosinophil percentages 18.3, 13.3, and 7 respectively. Liver panel showed mildly elevated transaminases, ALT 94 (7–45), and AST 65 (10–45).

Due to concern for recurrent PTLD refractory to chemotherapy and together with suspicion for malignancy, a decision was made to proceed with diagnostic laparoscopy with splenectomy and lymph node biopsy. However, owing to the patient’s thin habitus and limited domain due to gastrostomy tract, the spleen was difficult to mobilize and the decision was made to convert to an open splenectomy. This was successfully carried out.

Post-operative course was complicated by *Clostridium difficile* colitis for which she was treated as both inpatient and outpatient upon discharge. On postoperative day 7, she had met all discharge criteria and was therefore discharged home.

Pathology showed sections of right inguinal lymph node biopsy and spleen with multiple necrotizing and non-necrotizing granulomas consisting of central necrosis surrounded by palisading histiocytes/macrophages and multinucleated giant cells, with lymphocytes at the periphery of the granulomas. acid fast bacilli staining was negative for mycobacteria, GMS stain negative for fungi, and CD3 and CD20 negative, EBV stain was negative. Again, flow cytometry was did not show any evidence of non-Hodgkin lymphoma. Also, cytogenetic analysis did not show any clonal structural or numerical abnormality.

**Fig. 1:**
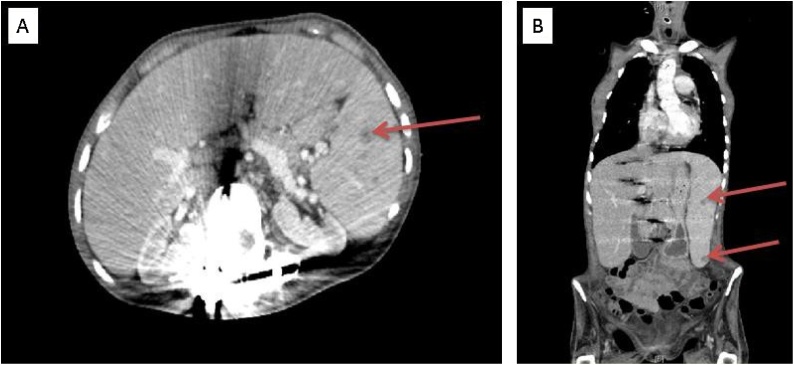
Computed tomography of the abdomen and pelvis with contrast and B shows the axial and coronal views respectively of computed tomography of chest, abdomen and pelvis demonstrating multiple ill-defined hypoattenuating lesions throughout an enlarged spleen.

**Fig. 2:**
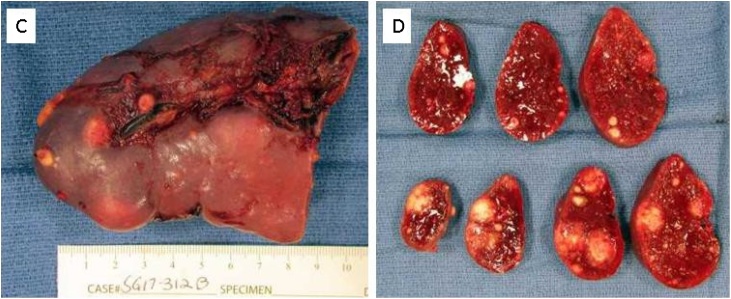
Gross spleen after splenectomy C) shows gross splenic capsular surface with necrotizing multinodular lesions. D) shows cut surface of the spleen with red parenchyma with numerous white nodules of varying sizes.

Our patient has encountered no complications from her splenectomy and no new lesions have been discovered on imaging as of April 2019. She continues on tacrolimus therapy for her cardiac transplant (Fig. 1, Fig. 2).

## 3 Discussion

A granuloma is defined as a focal, chronic inflammatory tissue response composed mostly of macrophages and their derivatives, evoked by unresolved, poorly degradable substances with a diagnosis based on histopathologic observations [[11], [12], [13]].

Necrotizing granulomas often indicate vasculitic lesions. In the necrobiotic granulomas the cellular infiltrate contains also neutrophils and eosinophils [14,15]. The presence of associated pathologic findings can also be useful in diagnosis. The examination of stained slides can aid in the correct diagnosis of the disease and serve as a valuable tool in retrospective analysis of archival material such as in our case. While the majority of necrotizing granulomas are infectious in etiology, around 25% of these findings remain unexplained [3]. The question as to why these granulomas occur without evidence of organisms is hypothesized to represent infectious granulomas in which microorganisms have been killed and/or removed by the inflammatory process [2].

Historically, PTLD associated with solid organ transplant has a mortality of 50% or higher [4,16]. Recurrence rates are not well documented due to the rarity of PTLD and associated high mortality rates. However, in a single center study by Rathore et al., a recurrence rate of 12% was reported [17]. The treatment of PTLD is not standardized and the clinical setting and concerns for risk of allograft rejection, associated comorbidities, or tumor burden often dictate the treatment strategy [18]. Nonetheless the treatment options available are RI, combination chemotherapy with or without rituximab, or surgery.

Reduction of immunosuppression (RI) has been the cornerstone of treatment for over 40 years in the treatment of PTLD. Despite its common usage, objective tumor responses to RI are inconsistent and highly variable ranging between 0–73% [[19], [20], [21], [22], [23]]. The long term prognosis is uncertain as the durable maintained response is seen in only 10–20% of cases [20,22,[24], [25], [26]]. In addition, the long median time to initial response of therapy and graft rejection/organ failure are two main concerns when considering RI for treatment of PTLD [27,23]. RI is not without risks and is not the ideal treatment for every patient. One factor that has shown to predict a poor outcome is multi-organ involvement. It has been shown that 40% of patients with multiorgan involvement of PTLD developed acute graft rejection [28]. Due to the high rate of rejection and multiorgan involvement of our patient, RI was deemed not a suitable therapy modality.

Another commonly used treatment of PTLD, especially when patients with PTLD do not respond to RI is the use of chemotherapy often combined with rituximab. Despite risks of toxicity and graft rejection, combined chemotherapy and RI has been found to be very effective with a complete response (CR) rate for PTLD up to 92% [29]. The most common chemotherapy regimen in the PTLD is CHOP (cyclophosphamide, doxorubicin, oncovin, prednisone). The CHOP regimen has been found to have a CR rate of 50%, and 5-year and 10-year progression-free survival (PFS) of 43% and 32% respectively [29]. Treatment guidelines on the use of RI versus chemotherapy have not yet been developed and treatment regimens are left to the discretion of the physician.

Surgery for localized recurrent or newly diagnosed disease may be beneficial. Surgical resection of PTLD is usually performed in conjunction with RI. In one study, 12 patients underwent surgical resection and RI. Of these patients there was no recurrence of PTLD identified after. 148 weeks of follow up, however, 7 of these patients had died from other causes unrelated to PTLD [30]. In the analysis, surgical resection in combination with RI was found to be superior to RI alone with a complete response rate of 74% compared to 63%, respectively [30]. Multiorgan PTLD, poor prognostic factors (increased age, elevated LDH ratio, severe organ dysfunction, presence of fever, night sweats and weight loss, and multi-organ involvement by PTLD at the time of diagnosis), or significant organ dysfunction are good indicators for the use of combined therapy including surgical resection [30]. While there are studies on effectiveness of combined surgical-RI therapy, there are no studies on the efficacy of adjuvant chemotherapy.

PTLD diagnosis requires histological analysis of tissue specimens/biopsies to be established. Since early lesions present in lymph nodes they are usually the first tissue to biopsy [31]. However, in a refractory PTLD, the spleen becomes the target tissue for histopathology analysis. In the setting of persistent PTLD with diffuse hypoattenuating splenic lesions on imaging diagnostic splenectomy becomes tissue sampling of choice although it carries more risks than percutaneous biopsy [32].

A large systematic review and meta-analysis of percutaneous image guided spleen biopsies compared the accuracy and complication rate percutaneous spleen biopsies to that of splenectomies. It was found that percutaneous spleen biopsies with needles smaller than 18 gauge demonstrate both a high accuracy rate with a specificity of 96.4% and low complication rate of 1.3% [32]. These results are similar to the accuracy and complication risk of liver or kidney biopsies. Percutaneous spleen biopsies were found to be a favorable alternative to splenectomy in cases where no other organ is available for biopsy [32].

PTLD is the most common cause of mortality in patients with solid organ transplant. While recurrent PTLD is rare, the high mortality of PTLD in solid organ transplant patients with history of PTLD should warrant a thorough evaluation. There are many risks associated with diagnostic work up for recurrent PTLD including, but not limited to, unnecessary diagnostic imaging with associated radiation exposure, invasive biopsies or possibly even surgery. Considering the high mortality associated with recurrent PTLD it is important to make correct diagnosis.

This paper is an attempt to make physicians aware of possibility of sterile granulomas in patients with PTLD when a lesion is stable but refractory to treatment while also highlighting the risk against the benefit of various diagnostic approaches when suspecting a PTLD splenic lesion. While percutaneous approach is minimally invasive there is always potential for misdiagnosis. Splenectomy is a safer approach considering high mortality associated with recurrent disease but it exposes patients to more invasive surgery and consequences of splenectomy. Advances in laparoscopic approach has allowed to perform minimally invasive splenectomies with very low complication rates and could be a potential avenue in borderline cases. Thus, cases with PTLD recurrence should be thoroughly evaluated with a multidisciplinary approach to provide the best diagnostic and management option for the patient.

## 4 Conclusion

Our case is unusual because of presence of sterile granulomas in patients with a history of PTLD. In a patient who is immunosuppressed, it would not be expected to find evidence of a robust immune response resulting in granulomas. Recurrent cases of PTLD are rare but due to the high mortality of PTLD it’s important to make a correct diagnosis. This case highlights existence of sterile granulomas as an entity in patients with a history of recurrent PTLD and hopefully encourages use of minimally invasive techniques to obtain an accurate diagnosis.

## Conflicts of interest

No financial or conflicts of interest.

## Sources of funding

Our case report was not funded and did not receive funding from any sources.

## Ethical approval

Our case report was exempt from ethical approval as it was a case report and therefore does not require an IRB approval.

## Consent

I, Nicholas Piening, assure the editors provide no alterations were made to distort scientific meaning while maintaining the confidentiality of our patient.

Written informed consent was obtained from the patient for this case report and accompanying images. A copy of the written consent is available for review by the Editor-in-Chief of this journal on request.

## Author’s contribution

Nicholas Piening: Study concept or design, writing the paper, literature review.

Saurabh Saxena: Study concept or design.

Salim Munoz Abraham: Study concept or design, writing the paper.

Hector Osei: Study concept or design, data/image collection.

Colleen Fitzpatrick: Study concept or design.

Jose Greenspon: Study concept or design, oversight and overview of the project.

## Registration of research studies

None.

## Guarantor

Jose Greenspon.

## Provenance and peer review

Not commissioned, externally peer-reviewed.
